# Categorization and Analysis of Primary Care mHealth Apps Related to Breast Health and Breast Cancer: Systematic Search in App Stores and Content Analysis

**DOI:** 10.2196/42044

**Published:** 2023-09-07

**Authors:** Sweekrity Kanodia, Jean Christophe Thalabard, Kevin Lhoste

**Affiliations:** 1 System Engineering and Evolution Dynamics Université Paris Cité, Inserm Paris France; 2 Learning Planet Institute Paris France; 3 Applied Mathematics Laboratory, MAP5, UMR CNRS 8145 Université Paris Cité Paris France

**Keywords:** breast cancer, breast self-examination, BSE, primary care, mobile applications, mobile apps, breast health, early diagnosis

## Abstract

**Background:**

Breast cancer is the most common cause of cancer mortality among women globally. The use of mobile health tools such as apps and games is increasing rapidly, even in low- and middle-income countries, to promote early diagnosis and to manage care and support of survivors and patients.

**Objective:**

The primary objective of this review was to categorize selected mobile health apps related to breast health and prevention of breast cancer, based on features such as breast self-examination (BSE) training and reminders, and to analyze their current dissemination. An ancillary objective was to highlight the limitations of existing tools and suggest ways to improve them.

**Methods:**

We defined strict inclusion and exclusion criteria, which required apps to have titles or descriptions that suggest that they were designed for the general public, and not for patients with breast cancer or health workers. Apps that focused on awareness and primary care via self-check were included, while those that focused on topics such as alternative treatments and medical news were excluded. Apps that were not specifically related to breast cancer were also excluded. Apps (in any language) that appeared in the search with keywords were included. The database consisted of apps from AppAgg and Google Play Store. Only 85 apps met the inclusion criteria. Selected apps were categorized on the basis of their alleged interactive features. Descriptive statistics were obtained, and available language options, the number of downloads, and the cost of the apps were the main parameters reviewed.

**Results:**

The selected apps were categorized on the basis of the following features: education, BSE training, reminders, and recording. Of the 85 selected apps, 72 (84.7%) focused on disseminating breast cancer information. BSE training was provided by only 47% (n=40) of the apps, and very few had reminder (n=26, 30.5%) and recording (n=11, 12.9%) features. The median number of downloads was the highest for apps with recording features (>1000 downloads) than those with education, BSE training, reminder, and recording features (>5000 downloads). Most of these apps (n=74, 83.5%) were monolingual, and around 80.3% (n=49) of these apps were in English. Almost all the apps on Google Play Store were free of charge.

**Conclusions:**

Although there exist several apps on Google Play Store to promote awareness about breast health and cancer, the usefulness of most of them appears debatable. To provide a complete breast health package to the users, such apps must have all of the following features: reminders or notifications and symptom recording and tracking. There is still an urgent need to scientifically evaluate existing apps in the target populations in order to make them more functional and user-friendly.

## Introduction

**Figure 1:**
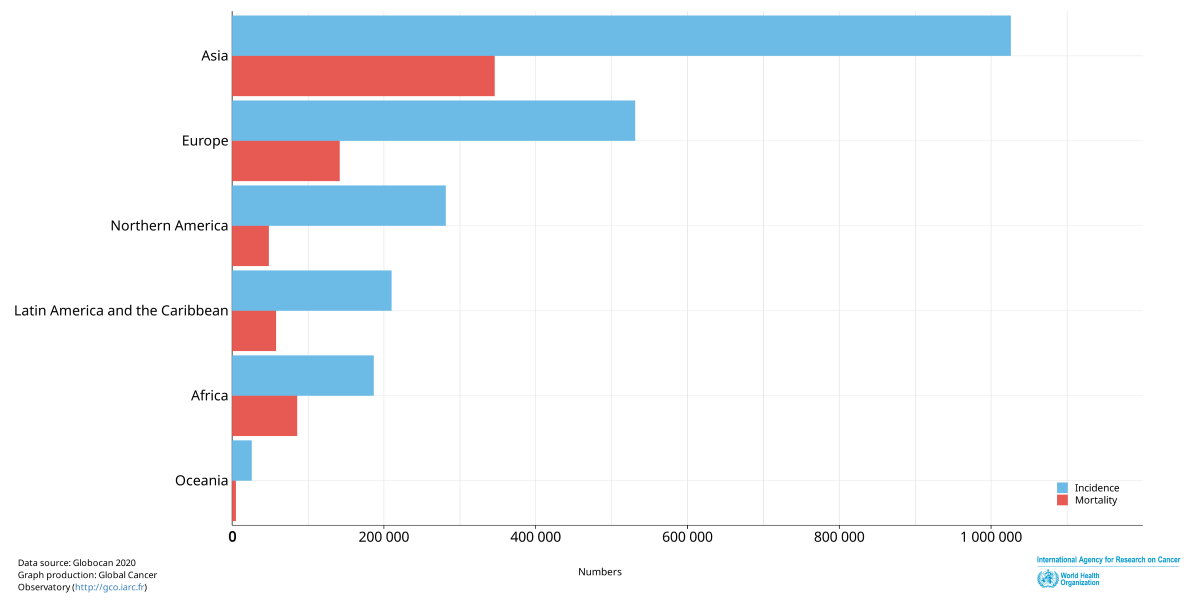
Estimated number of incident cases and deaths due to breast cancer in women of all ages in different continents. Data were obtained from the International Agency for Research on Cancer [4].

According to an estimation by the International Agency for Research on Cancer in 2020, every minute, about 4 women are diagnosed with breast cancer, and 1 dies of it. Breast cancer is the most common cancer among women and the most common cause of cancer-related mortality (Tables 1 and 2) [1]. Trends have changed significantly over the past few years with more than half of incident cases and deaths due to breast cancer occurring in low- and middle-income countries, which represent 80% of the world’s population [2] in comparison to high-income countries [3]. Asia contributes to 45.4% (n=1,026,171) of the new breast cancer cases among the total of 2,261,419 and 50.5% of related deaths (Figure 1) [4].

The World Health Organization’s (WHO’s) Global Breast Cancer Initiative aims to reduce global breast cancer mortality by 2.5% per year [5]. To meet this goal, the WHO identified 3 pillars that are important for reducing breast cancer–related mortality: awareness to promote early diagnosis, proper screening programs for timely diagnosis, and comprehensive breast cancer management [5]. From a pragmatic point of view, strengthening the screening system and enforcing nationwide comprehensive management essentially depend on women’s awareness of breast cancer.

David Forman [3], head of the International Agency for Research on Cancer’s Section of Cancer Information, said, “Breast cancer is also a leading cause of cancer death in the less developed countries of the world. This is partly because a shift in lifestyles is causing an increase in incidence, and partly because clinical advances to combat the disease are not reaching women living in these regions.”

A survey carried out in Kathmandu Institute of Science and Technology, Kathmandu, Nepal, revealed that 70% of Nepalese women had never heard of breast cancer [6]. Another study carried out in Pokhara, Nepal, reported that the knowledge of breast cancer symptoms is very poor [7]. More than 50%-80% of patients with breast cancer in India, 49% of those in Karachi (Pakistan), and 47% of those in Iraq are diagnosed at advanced stages due to poor awareness and poor screening programs [8,9]. Overall, 62% of breast cancer deaths are due to presentation at advanced stages in low- to middle-income countries [2]. Furthermore, several studies reflect better survival rates among educated women [10-15].

According to Gadgali et al [16], “This emphasizes the need for breast awareness programmes and educational material to be delivered in various local and regional languages in order to reach the less as well as uneducated and underserved women, in order to improve their survival.”

Periodic screening, by regular self-checks or through imaging modalities including ultrasonography and mammography, is important in early diagnosis of breast cancer and other breast conditions. Breast self-examination (BSE) is a free and the most convenient modality as it is painless and can be done by oneself without any special equipment or tools. It has been incorporated into international cancer control programs targeting economically disadvantaged low- and middle-income countries [2] as an important tool for early diagnosis. Tara et al [17] reported 68% congruence in findings from BSE carried out by participants and clinical examination performed by health experts. However, BSE results in a high number of false positives and generates anxiety in women.

To tackle the discussed gaps in knowledge regarding breast cancer, its risk factors, and its symptoms, screening programs including BSE and the promotion of both awareness and education are needed. Additionally, providing women with proper tools to learn about BSE or training them will aid in efficiently tackling the potential associated anxiety. Developments in mobile health (mHealth) and growing internet connectivity might contribute to providing appropriate tools to help achieve these goals.

mHealth is broadly defined as mobile and internet-based interventions for health purposes. It can encompass various forms including guidelines, tutorials, games, visual novels. With increasing use of mobile phones and the internet [18], mHealth is now being used in different countries including India [19] and Ethiopia [20] to improve access to health. Success stories, such as that reported by Lin et al [21] about Quit Genius, provide a strong basis for how an mHealth app might result in behavioral changes. Lin et al [21] reported that 36% of Quit Genius users successfully quit smoking, and 59.6% of them reduced the number of cigarettes they smoked per day [21].

There exist several apps on mobile app stores such as Google Play Store and Apple App Store, which aim to promote knowledge about breast cancer. Etege [20] is one such example, designed to aid Ethiopian women in performing BSE and recording symptoms. It has now reached millions of women. Dear Mamma [22] is another very popular breast health app developed by the DEAR Foundation, Switzerland, which teaches women about BSE and allows recording symptoms and setting monthly reminders. Unfortunately, only 13% [23] of all the available breast health–related apps focus on promoting awareness among all women, and these apps have not been scientifically evaluated.

This paper reviewed breast health mobile apps that include features such as BSE training, symptom recording, reminders, and information and educational guides. Categorization of such apps, available to women for self-education about breast health, is also reviewed here. Breast health can be defined as keeping breasts healthy by preventing breast cancer and other benign conditions. Good breast health requires women to be aware of not only symptoms of breast cancer but also other benign conditions. It includes knowledge of BSE, breast cancer diagnosis, and treatment of and correlation among factors such as smoking, alcohol intake, contraceptives, breastfeeding, age at first childbirth, menarche, and menopause, which may affect breast health.

Organizations such as the WHO that are now focusing on using mHealth to promote awareness could also benefit from this study. They could also use this list to choose befitting apps that could be used as educational interventions to educate women about breast cancer. In general, the data collected and analyzed in this study will help in the advancement of mHealth apps related to breast health and result in better apps in the future.

**Table 1:** Number of new cancer cases among women (according to the 2020 Global Cancer Observatory of the International Agency for Research on Cancer; N=9,227,484).

Cancer type	Individuals, n (%)
Breast	2,261,419 (24.5)
Colorectal	865,630 (9.4)
Lung	770,828 (8.4)
Cervix uteri	604,127 (6.5)
Thyroid	448,915 (4.9)
Corpus uteri	417,367 (4.5)
Stomach	369,580 (4)
Other types	3,489,618 (37.8)

**Table 2:** Number of cancer deaths among women (according to the 2020 Global Cancer Observatory of the International Agency for Research on Cancer; N=9,227,484).

Cancer type	Deaths, n (%)
Breast	684,996 (15.5)
Colorectal	419,536 (9.5)
Lung	607,465 (13.7)
Cervix uteri	341,831 (7.7)
Liver	252,658 (5.7)
Pancreas	219,163 (4.9)
Stomach	266,005 (6)
Other types	1,637,669 (37)

## Methods

### App Search and Data Extraction

**Figure 2:**
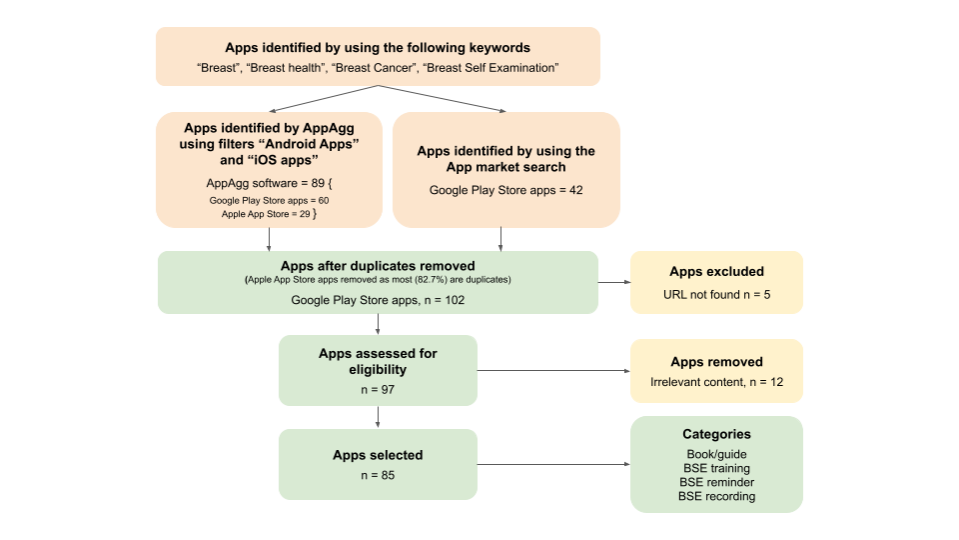
A modified PRISMA (Preferred Reporting Items for Systematic Reviews and Meta-Analyses) flowchart adapted to extensive review of mobile apps. BSE: breast self-examination.

An extensive search was conducted on Google Play Store, Apple App Store, and the AppAgg tool using the keywords “Breast,” “Breast health,” “Breast Cancer,” and “Breast Self-Examination.” Search words were limited to English, keeping in mind that the authors’ proficiency is poor in other languages. However, all apps that appeared using these search words were analyzed regardless of the language of the app by translating the content using Google Translate. The PRISMA (Preferred Reporting Items for Systematic Reviews and Meta-Analyses) guideline [24] was modified and adapted to guide this review (Figure 2).

Apps were selected using the approach of Bender et al [25], and stringent inclusion and exclusion criteria were defined (see Textbox 1). Apps were selected on the basis of either primary education or early diagnosis (via BSE; or both), including breast cancer guides, BSE training, reminders, and symptom recording. All the other apps (including those focusing on breast workouts, breast shape photo editors, breastfeeding, breast pumping, breast surgery or implants, and breast cancer care) were excluded.

A first round of app search was carried out on AppAgg [26]. AppAgg is a free software that has accumulated large amounts of data about the apps available on all app stores such as Google Play Store, Apple App Store, and Windows. Apps on Google Play Store (n=60) were selected by applying the filter “Android apps” and using the following search keywords: “Breast,” “Breast health,” “Breast Cancer,” and “Breast Self-Examination.” Similarly, apps on Apple App Store (n=29) were selected by applying the filter “iOS apps” and using the abovementioned search keywords.

AppAgg provides data in the format shown in Textbox 2. Of these, information regarding the version, developer, subcategory, updates, and release date were removed because it was not informative and of no importance to the study. Information regarding in-app purchase, new, preorder, rating, and vote were discarded because of missing information (97%, 99%, 100%, and 100%, respectively).

Information regarding name, price, category, and downloads was verified manually one by one by comparing it with data available on Google Play Store and Apple App Store. The number of downloads was updated during this process. Additionally, language options available were included while updating information from AppAgg.

A second round of searches using keywords was carried out directly on Google Play Store and Apple App Store. An additional 42 apps from Google Play Store were included in the list. Information regarding name, price, category, and downloads about these additional apps was also recorded in an Excel (Microsoft Corp) spreadsheet.

In total, 131 potential apps were identified (as of October 8, 2021): 29 on Apple App Store and 102 on Google Play Store based on their descriptions and screenshots.

**Inclusion and exclusion criteria met while selecting the apps.**

**Inclusion criteria**

- Apps that appear using the keywords
- Apps can be in any language or can support multiple languages
- The title or description (or both) suggests that the app is related to breast cancer or breast self-examination
- The title or description (or both) suggests that the app has features including educational content (about breast cancer and breast health), self-check reminder, symptom recording (such as notes), or tracking (or history of recorded symptoms)

**Exclusion criteria**

- The title or description (or both) suggests that the app is related to other cancers or to cancer in general
- The title or description (or both) suggests that the app is related to education on alternative therapy, food habits to prevent cancer, or breast enhancement strategies
- The title or description (or both) suggests that the app is related to risk assessment questionnaires only and serves no other purpose
- The title or description (or both) suggests that the app is related to care management, survivorship, and postcancer standard of living for patients with breast cancer
- Apps intended for patients, health care providers, and caregivers, which track and monitor breast cancer or provide support and care to patients with breast cancer
- Google Play Store apps were retained if they were available on both Google Play Store and Apple App Store

**Data extracted using AppAgg.**

- Number
- Name
- Version
- Price
- In-app purchase
- Developer
- Category
- Subcategory
- New
- Downloads
- Updated
- Released
- Preorder
- Rating
- Vote
- Store ID
- URL

### Data Refining

Based on app descriptions and screenshots available on the marketplace, 131 apps meeting the inclusion criteria were shortlisted. Google Translate was used to translate the descriptions of the apps if they were in a language other than English. Screenshots of the apps on the app marketplaces (Google Play Store and Apple App Store) also provided information about the features of the apps. In case of poor descriptions and no screenshots, apps were downloaded on a OnePlus 7 device and studied (author SK). These apps were further independently reviewed by author JCT, an MD qualified in gynecology and breast disease, and author KL, a computer scientist with expertise in the digital space, to ensure reliability of the study.

Apps on Apple App Store (n=29) were excluded from the list for the following reasons: (1) use of Android phones is much more widespread than the use of iPhones, (2) most (n=24, 82.7%) of these apps are on Google Play Store and would lead to the inclusion of duplicates, and (3) not much information could be gathered about apps on Apple App Store.

Five out of the remaining 102 apps were excluded as their URL could not be found when reviewed by the 2 reviewers JCT and KL. Twelve apps were further excluded as they were focused on nutrition, alternative therapy, including naturopathy for breast cancer and Ayurveda, and risk assessment surveys or contained frequently asked questions about innovations in treatment and cure—they were considered irrelevant by the reviewers as they did not meet the inclusion criteria.

The remaining 85 apps (highlighted in Multimedia Appendix 1) that met the inclusion criteria were further studied and categorized on the basis of their alleged interactive features.

### Categorization of the Selected Apps

To the best of our knowledge, there are no prior reviews on mHealth apps for primary care and awareness of breast health and breast cancer. Hence, we identified features based on which the apps were categorized, in accordance with our understanding and need for primary care and education.

Four important interactive features were identified on the basis of which apps were categorized, namely education (E), BSE training (T), BSE reminders (R), and BSE symptom recording (Re), as shown in Table 3. Some of these features including reminders, BSE training, and education have been recently reported by Nasution et al [27] as important while developing mobile apps for breast health. BSE recording was added as it was deemed important since it helps users keep a note of changes in their breasts.

The E category includes content and information about risk factors, prevention, symptoms, treatment, diagnosis, benign conditions of breasts, or BSE. Category T refers to step-by-step tutorials about how to self-examine breasts via videos, pictures, or text. Category R implies that the app allows users to select a date for monthly check and notifies users on that date every month. Category Re refers to apps that allow users to take notes of changes observed during self-check via audio, video, or text. “Holistic apps” in this study are defined as apps that contain all the discussed features and are categorized as “E,T,R,Re.”

The 85 apps were divided into 10 different categories as described in Table 4 (see Multimedia Appendix 1 for a complete list of apps after categorization). The “category” column was replaced by these functional categories as shown in Table 4. Two independent reviewers analyzed the categorization on the basis of the narrative text corresponding to the description of each app and screenshots provided by Google Play Store. Final categorization was achieved through mutual agreement between the 2 reviewers.

**Table 3:** Four alleged interactive features used to define the apps (for simplicity in writing, codes are allocated to the different features).

Features	Code	Description
Education	E	Educational—promotes knowledge about breast cancer, symptoms, risk factors, screening and treatments, and benign conditions via text and images
BSE^a^ training	T	BSE step-by-step training
BSE reminder	R	Reminders and notifications for monthly BSE
BSE symptom recording	Re	Feature to record symptoms found

^a^BSE: breast self-examination.

**Table 4:** Categorization of the 85 apps based on the 4 features discussed in Table 3.

Categories	Code	Description
Education	E	Apps that have educational content about breast cancer, symptoms, risk factors, screening and treatments, and benign conditions via text and images
BSE^a^ training	T	Apps that have BSE step-by-step training
BSE recording	Re	Apps that only allow recording of symptoms and evaluation of risk from them
Education and BSE training	E, T	Apps that have features including educational content and BSE training
Education and BSE reminder	E, R	Apps that have features including educational content and reminders or notifications
BSE training and reminder	T, R	Apps that have features including BSE training and reminders or notifications
Education, BSE training, and reminder	E, T, R	Apps that have educational content, BSE training, and reminders or notifications
Education, BSE training, and recording	E, T, Re	Apps that have educational content, BSE training, and symptom recording
BSE training, reminder, and recording	T, R, Re	Apps that support BSE training, symptom recording, and reminders or notifications
Education, BSE training, reminder, and recording	E, T, R, Re	Apps that have all the identified interactive features

^a^BSE: breast self-examination.

### Statistical Analysis

SPSS (version 29; IBM Corp) was used to analyze descriptive statistics of each of the following parameters: number of relevant apps available on Google Play Store versus those available on Apple App Store, primary characteristics or features, number of downloads, language options, and cost. The median number of downloads was also evaluated against the discussed features and categories; median values were calculated since the data were skewed. For analysis, “Verbose” is defined as the number of words exceeding 20 per sentence in a corpus of text (per the Institute of Medicine’s [IOM’s] guidelines for Health Literate Apps [28]).

## Results

### Google Play Store or Apple App Store? Which Marketplace Has More Related Apps?

It was interesting to note that there are approximately 300% more related apps on Google Play Store than on Apple App Store. Additionally, 82.7% (24/29) of apps on Apple App Store were also available on Google Play Store.

### Primary Characteristics or Features of the Apps

The selected 85 apps were categorized on the basis of their features (as shown above).

As evident from Table 5, most of the apps (n=72, 84.7%) had educational content for users to learn about breast cancer, early diagnosis, treatment, and symptoms. All the E apps exceeded 25 words per sentence, not adhering to the IOM’s guidelines for Health Literate Apps [28].

Less than half of the selected apps (n=40, 47%) focused on BSE training. Very few of them had additional features such as reminders and notifications for monthly scans (n=26, 30.5%) and “notes” features to record symptoms (n=11, 12.9%). Only 7 (8.2%) apps incorporated together all the discussed features (those categorized as “E,T,R,Re”).

**Table 5:** Number of apps per category (as explained in illustrating how features are distributed among the studied apps.

Category	Apps, n
Education	42
BSE^a^ training	4
BSE recording	1
Education and BSE training	8
Education and BSE reminder	2
BSE training and reminder	7
Education, BSE training, and reminder	11
Education, BSE training, and recording	2
BSE training, reminder, and recording	1
Education, BSE training, reminder, and recording	7

^a^BSE: breast self-examination.

### Number of App Downloads

Most of the apps (82.4%) had only >1000 downloads, as evident from Table 6. As depicted in Table 7, apps categorized as “E,T,R,Re” had the highest median number of downloads. The median was also extracted from the number of downloads of all the apps with the discussed features: “E,” “T,” “R,” and “Re.” Apps with the Re feature had the highest number of median downloads (Table 8).

One of the apps that had the highest number of downloads was “Breast Cancer Guide”—an E app that promotes knowledge about primary care for breast cancer via videos. Only one app (Dear Mamma) out of 3 apps with >50,000 downloads was a holistic app (E,T,R,Re) with all the discussed interactive features.

**Table 6:** Number of downloads of the selected apps as determined by Google Play Store.

Number of downloads	Apps, n
1000	70
5000	7
10,000	5
50,000	3

**Table 7:** Median number of downloads of all apps containing the following features: education, breast self-examination (BSE) training, BSE reminders, and BSE recording.

	Number of downloads, median
Education	500
BSE training	750
BSE reminders	1000
BSE recordings	300

**Table 8:** Median number of downloads of all apps based on their categories.

	Number of downloads, median
Education	300
BSE^a^ training	275
BSE recording	50
Education and BSE training	1000
Education and BSE reminder	5.5
BSE training and reminder	100
Education, BSE training, and reminder	500
Education, BSE training, and recording	1000
BSE training, reminder, and recording	1
Education, BSE training, reminder, and recording	5000

^a^BSE: breast self-examination.

### Language Options Available

As clearly depicted in Multimedia Appendix 2, overall, 83.5% (n=71) of the selected apps were monolingual; of them, 80.3% of apps were in English. Only 7.1% of them were multilingual (>5 languages). Apart from English, other common languages in these apps were Arabic, Spanish, German, French, Bangla, and Hindi (Multimedia Appendix 3).

Only 17.08% of people worldwide speak English natively or as a second language [29]. Yet, the most commonly used language in these apps is English (71.8%; Table 8). The Dear Mamma app has 11 language options available and is one of the few apps to have >50,000 downloads.

### Cost of the Selected Apps

All of the selected apps on Google Play Store were free of charge.

## Discussion

### Principal Findings

We conducted a systematic internet search of the existing breast health apps. Five key features of these apps were defined, representing their level of primary care and awareness. A total of 85 apps on Google Play Store were identified and analyzed. Based on the defined features, these apps could be classified into 10 different categories.

Most of the apps (n=72, 84.7%) were clearly educational, containing mainly text (when evaluated against IOM guidelines for Health Literate Apps). The Breast Cancer Guide app, with the highest number of downloads, used videos to provide information; this indicates that videos might be a good way to engage users. Surprisingly, less than half of them (n=28, 38.9% of E apps, accounting for 40, 47% of all apps) had content explaining BSE. Since most of these apps were monolingual (n=71, 83.5%) and in English (80.3% of monolingual apps, 71.8% of all apps), their use remains restricted to women literate in English. Limited accessibility can also be inferred from their rather low downloads (n=70, 82.4% of apps with less than 1000+ downloads). Tables 7 and 8 show that apps categorized as “E,T,R,Re” have the highest number of median downloads and those categorized as “Re” have the highest number of median downloads. This could indicate that users have a selection bias toward apps that help them record their symptoms alongside having BSE training, reminders, and educational material. Unfortunately, only 8.2% (n=7) of apps are holistic (E,T,R,Re) and only 12.9% (n=11) of all apps have the Re feature.

With more than 3 million apps on Google Play Store [30,31] and 2 billion active users [32,33], Android has captured the app market. Most apps on Apple App Store (n=24, 82.7%) were already identified on Google Play Store and studied. Therefore, we decided to focus our study on apps (n=85) available in Google Play Store. Most of the apps were designed to promote knowledge about breast cancer and breast health. Only 40 (47%) apps had visual or textual information about BSE, serving as essential and accessible tools for women to proactively care for their breasts.

Additionally, information about the appropriate timing to perform self-examination in relation to the menstrual cycle, that is, 3 to 5 days after the start of menstruation [34,35], needs to be highlighted in these apps. The reminder feature can be improved through synchronization with period tracking apps. With information regarding period start date at the forefront, selecting dates for monthly BSE will be easier and automatic, resulting in a reliable outcome on BSE.

Another very important addition to these apps can be a well-defined symptom recording feature with all the symptoms enlisted as images or graphics. This will make it easier for users to select their symptoms, if any, especially for low-literacy audiences. Low-literacy populations can also benefit from apps in regional languages with audio aid, for example, integration of text-to-speech modalities such as Siri or Alexa.

### Limitations

There are some limitations that need to be discussed. As we observed, most apps were in English. It can be debated that the bias toward English is due to the use of English keywords. However, it is clear from the list that apps in languages other than English were also obtained using these keywords. This can be attributed to the English keywords used by developers while uploading their apps on Google Play Store. To cross-validate, we ran a search with the keywords translated in Mandarin (Chinese) and Spanish (two of the most spoken languages worldwide [36]) and no extra apps were recorded.

“Number of downloads” displayed on Google Play Store indicates the number of unique downloads by different users. Use of “number of downloads” to assess dissemination of the apps might be insufficient. The limitation of this metric is that it does not consider downloads on different devices by the same user (same login ID). Other key retention metrics such as “monthly active users” might be a better proxy.

A general limitation of studies on mHealth apps is that we do not very well understand how the App Stores evaluate apps on their marketplace. Very little to no information is available about that. Also, apps on these marketplaces are not stable. New apps are continually added, and some apps are removed under several circumstances including restricted content, duplication, intellectual property fraud, privacy issues, or if developers choose to remove their app. Thus, reviews on mHealth apps cannot be replicated easily. When we ran our search again, we found the same number of results, but 4 apps in the list were different.

### Future Prospects

mHealth apps are increasingly improving the standard of care and health, especially in poor, rural countries such as certain African countries, Nepal, and Bangladesh [37,38]. In countries such as Nepal, most women live in remote, rural areas and do not have access to diagnostic centers, clinics, or hospitals. A minimum of 3 hours of travel [39,40] are required to make a visit to the closest clinic or hospital, which, again, may or may not be well equipped. Breast health apps might provide support to women by aiding them in periodic checks of their breasts [41,42]. Regular breast checks might result in identification of smaller tumors early, resulting in better treatment options and faster recovery.

As we have discussed in this study, current apps are not very user-friendly since they are verbose, have few language options, and lack important features. Customized content factoring in cultural and linguistic differences has been shown to improve accessibility and participation in breast cancer screening [43-45] and might be useful in designing apps for primary care. Well-designed user experience research, followed by a participatory approach for app development (with target audience) [46-48] might help bridge the gap between such apps and their users.

Another approach to make apps more interesting would be gamifying them [47-51]. Gamification is defined as adding game mechanics to nongame environments such as websites and apps. To the best of our knowledge, no breast cancer apps have incorporated game elements yet.

A new direction of research could be the evaluation of mHealth apps. These apps, although useful in primary care, do not seem to have been properly evaluated. There is a need for standard guidelines and protocols [28] that ensure proper evaluation, including those for the design, content, and language of such apps. Prior to launch, pilot and validation studies of these apps might further ensure acceptability and adaptability. These apps can then be usefully integrated into the health care system in the future, seeking to improve the standard of care remotely.

## Appendix

Multimedia Appendix 1 List of selected apps that have been categorized as discussed in Table 4.

Multimedia Appendix 2 Mono-, bi-, tri-, or multi- lingual: the number of languages available on the selected apps. Color gradient from Dark to Light indicating Monolingual to multilingual apps.

Multimedia Appendix 3 Percentage of selected apps available in different languages, namely, English, Hindi, German, French, Spanish, Bangla, Arabic and others. Color gradient from dark to light indicating most used language to the least used.

## Abbreviations

BSE: breast self-examination
E: education
IOM: Institute of Medicine
mHealth: mobile health
PRISMA: Preferred Reporting Items for Systematic Reviews and Meta-Analyses
R: breast self-examination reminder
Re: breast self-examination recording
T: breast self-examination training
WHO: World Health Organization

## Data Availability

All data generated or analyzed during this study are included in this published article as Multimedia Appendix 1. Data acquisition was concluded on October 8, 2021.
