# Supplementary figures and images for: Categorization and Analysis of Primary Care mHealth Apps Related to Breast Health and Breast Cancer: Systematic Search in App Stores and Content Analysis
Source: JMIR Cancer. 2023 Sep 7;9:e42044. doi: 10.2196/42044 (PMC10514767; doi:10.2196/42044)

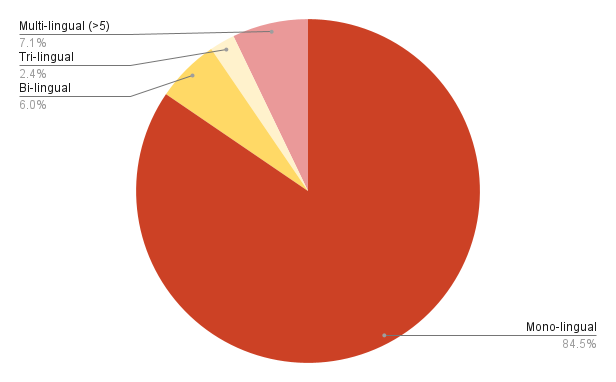

Supplement: Multimedia Appendix 2 [file cancer_v9i1e42044_app2.png]

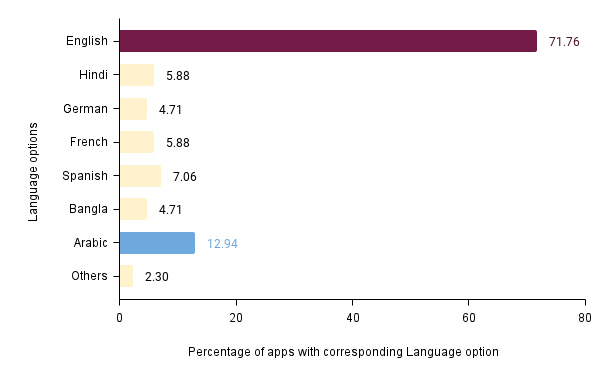

Supplement: Multimedia Appendix 3 [file cancer_v9i1e42044_app3.png]
